# Pre-copulatory choices drive post-copulatory decisions: mechanisms of female control shift across different life stages

**DOI:** 10.1186/s12862-023-02138-6

**Published:** 2023-06-27

**Authors:** Lenka Sentenská, Catherine E. Scott, Luciana Baruffaldi, Maydianne C. B. Andrade

**Affiliations:** 1 Department of General and Systematic Zoology, University of Greifswald, Loitzer Strasse 26, 17489 Greifswald, Germany; 2 Department of Biological Sciences, University of Toronto Scarborough, 1265 Military Trail, Toronto, ON M1C 1A4 Canada; 3 Department of Natural Resource Sciences, McGill University, 21111 Lakeshore Road, Ste-Anne-de-Bellevue, Montréal, Québec H9X 3V9 Canada

**Keywords:** Immature mating, Female mate choice, Wallflower effect

## Abstract

**Background:**

The ‘wallflower’ hypothesis proposes females mate indiscriminately to avoid reproductive delays. Post-copulatory mechanisms may then allow ‘trading up’, favouring paternity of future mates. We tested links between pre- and post-copulatory choice in *Latrodectus geometricus* female spiders paired sequentially with two males. These females copulate as adults or as subadults and store sperm in paired spermathecae. Choosy adults have a higher risk of delays to reproduction than subadults.

**Results:**

We predicted low pre-copulatory, but high post-copulatory choice at first matings for adults and the opposite for subadults. At second matings, we expected all females would prefer males superior to their first. We found all females mated indiscriminately at their first pairing, but in contrast to subadults, adults usually allowed only a single insertion (leaving one of their paired spermatheca empty); a mechanism of post-copulatory choosiness. Adult-mated females were more likely to remate than subadult-mated females when they became adults, showing a preference for larger males, while subadult-mated females tended to prefer males of greater size-corrected mass.

**Conclusions:**

Our results show that the ‘wallflower’ effect and ‘trading up’ tactics can be utilized at different life stages, allowing females to employ choice even if rejecting males is costly.

**Supplementary Information:**

The online version contains supplementary material available at 10.1186/s12862-023-02138-6.

## Background

Reproductive fitness depends on a sequence of episodes of selection, where each may operate based on independent criteria, and may be linked to, or influence, other episodes [1]. As our understanding of pre- and post-copulatory processes expands, so does understanding of how the interplay of decisions at each stage may affect overall fitness [2]. Females may impose selection on males through choice based on male phenotype [3, 4]. Choice may manifest through variation in the propensity to mate, or through female-controlled variation in paternity of mates through behavioural or physiological mechanisms [5]. The expression of a preference that involves rejecting a courting male (pre-copulatory choice) may be affected by a range of internal and external factors that determine the fitness benefits of choosiness for a female [6, 7]. Post-copulatory processes associated with that first mating may not align with pre-copulatory choice, however, as the decision of whether to mate may diverge from the decision to employ mechanisms to control paternity of that first mate. Similarly, when a previously-mated female is courted by a new male, the factors affecting the decision to remate may shift relative to the first mating. For example, after copulation with one male, females may no longer risk infertility or delays to reproduction by rejecting males, so choosiness may increase [8, 9]. In addition, rather than an absolute phenotypic threshold, females may compare a courting male to the first mate, and only remate if the second male is superior along some phenotypic axis (e.g., ‘trading up’, [10–12]).

Selection is expected to favour female choice when male traits vary and when offspring fitness is linked to paternal traits [3, 13]. Common characteristics subject to female choice include courtship displays, male ornaments, size or condition [3]. In addition to assessing favoured traits, understanding patterns of choice also requires mapping out choosiness, that is, the likelihood of expressing a preference [8]. For females, pre-copulatory choosiness may decrease in response to various types of costs of choice, including predation risk [14], male-imposed costs of resisting mating attempts [15], and for unmated females, the risk of delays to reproduction [9, 16]. There is variation in whether these factors would also affect the expression of post-copulatory choosiness, and costs may shift between the pre- and post-copulatory contexts. However, in situations where choice is favoured, but constrained by pre-copulatory costs, we might predict a stronger appearance of post-copulatory mechanisms that allow females to ‘trade up’ to higher quality males encountered after their first mating [e.g., 12]. Support for the ‘trade up’ hypothesis has included demonstrations that female mating decisions depend on the phenotype of the second male relative to the first, higher paternity of attractive second males [12], or that post-copulatory mechanisms result in an increased opportunity for a second male to acquire paternity [17, 18]. Currently, there are few tests that include assessment of the interplay between mating, remating, and mechanisms of post-copulatory choosiness. Here we examine these patterns in concert, asking whether females use pre-copulation and post-copulation mechanisms as predicted to favour males of particular phenotypes in a species where ‘wallflower’ effects are predicted to lead to low choosiness in the first mating interaction [e.g., 18, 19].

We studied the brown widow spider (*Latrodectus geometricus*), one of three species of *Latrodectus* in which females can mate not only as adults but also as subadults several days prior to their final moult to maturity (‘immature mating’; [20, 21]). The sperm is retained during the moult to adult stage and so once subadult-mated females reach maturity, they produce similar numbers of viable offspring (*L. geometricus, L. hasselti;* [20, 22]) or more offspring (*L. hesperus*; [21]) than females that mate only as adults. Adult and subadult courtship and mating sequences may differ (*L. hasselti, L. geometricus, L. hesperus*, [21, 23]), as do post-copulatory mechanisms that could affect paternity if females remate. Female *Latrodectus* spiders have paired sperm storage organs (i.e. spermathecae) and males paired copulatory organs and so males must achieve two insertions to inseminate both spermathecae. Additionally, during each insertion, males may also place a sperm plug in each spermatheca. The plug consists of a broken portion (i.e. apical sclerite) of one of the male’s two reproductive organs that blocks insemination by subsequent males when placed correctly (a feature of mating across many *Latrodectus* species; [24]). The frequency of successful plug deposition varies, with estimates between 55% and 90% in the species where it has been studied (*L. tredecimguttatus*, [25]; *L. hasselti*, [18, 26]; *L. pallidus*, [27]; *L hesperus*, [28]; also see [29]). Successful plugs in both spermathecae mediate first male sperm precedence, but if a male inseminates and successfully plugs only one spermatheca he will share paternity if the female remates [26]. Although the mechanism is unclear, there is evidence that cues of male availability lead females to block plug placement as predicted by the wallflower hypothesis (in *L. hasselti* [18]) using some internal mechanism that is as yet unknown, but may be a feature of the internal anatomy of *Latrodectus* females [24.] Thus females may control paternity of rival males through several mechanisms, including mating success, insertion number within each mating, or successful plug placement (reviewed in [24]).

When female *L. geometricus* mate as adults, males engage in lengthy courtship, and during copulation their bodies are twisted into the female’s mouthparts in a ‘copulatory somersault’ [30]. This behaviour often triggers a cannibalistic attack, which may occur during the first or second insertion. In contrast, when mating with subadult females males court only briefly and do not perform copulatory somersaults. Since the copulation is not interrupted by female cannibalistic attacks, males are typically able to insert both copulatory organs and plug both spermathecae [20]. The cuticular lining is not shed with the rest of the cuticle during the final moult, so plugs deposited during immature mating are retained in the adult stage [31]. Moreover, although not examined in *L. geometricus*, studies in a number of other *Latrodectus* species across different continents incl. species from similar climatic and seasonal climatic environments [32] suggest there is a significant risk to pre-copulatory choice for adult females, due to variation in sex ratio and population density in the field, and attendant risk and costs of delays to mating [e.g., 24, 33, 34]. In two species, females respond to cues of low male density by reducing choosiness in their first encounter, which would reduce the risk of remaining unmated if encounters with additional males are unlikely (*L. hasselti*: [18]; *L. hesperus*: [19]). This may be one of the reasons for selection on adult females to employ post-copulatory mechanisms of choice [24]. It is not clear whether the same cost-benefit balance applies for subadult females, as some previous studies indicate that subadult females are less likely to mate with the first male that courts [20, 35, but see 36] and that immature matings often result in both sperm storage organs being plugged by the first-mating male [20, 31].

In this study, we expose immature and mature females to two males in sequence. We assess potential female deterrent behaviours [e.g., 23], pre-copulatory cannibalism, mating frequency and timing as measures of female pre-copulatory choosiness, and mating outcomes (no. of insertions, frequency and timing of sexual cannibalism, sperm plug placement) as measures of post-copulatory choosiness. We then ask whether adult females first mated as adults (‘adult-mated females’) or as subadults (‘subadult-mated females’) differ in remating behaviour and post-copulatory outcomes, as a function of first-mating patterns and the relative phenotypes of the first and second males. Following the ‘wallflower’ hypothesis [9], we predicted that adult females would mate indiscriminately in the first pairing to avoid the risk and cost of mating delays or failure, but that they would employ mechanisms that enable post-copulatory choice. We then expected them to ‘trade-up’ by remating with second suitors whose phenotypes are sufficiently superior to those of their first mate. In contrast, we expected that subadult females would be choosier at their first mating opportunity, because they do not face the same risk of delays to reproduction (they must wait until maturity to produce egg sacs) and encounters with males during development provide cues of high mate availability [18, 19]. We also predicted that subadult-mated females would be more likely to remate upon maturity than adult-mated females, given the relatively limited investment by males into courtship with subadult females.

## Results

### First mating trial

**Fig. 1:**
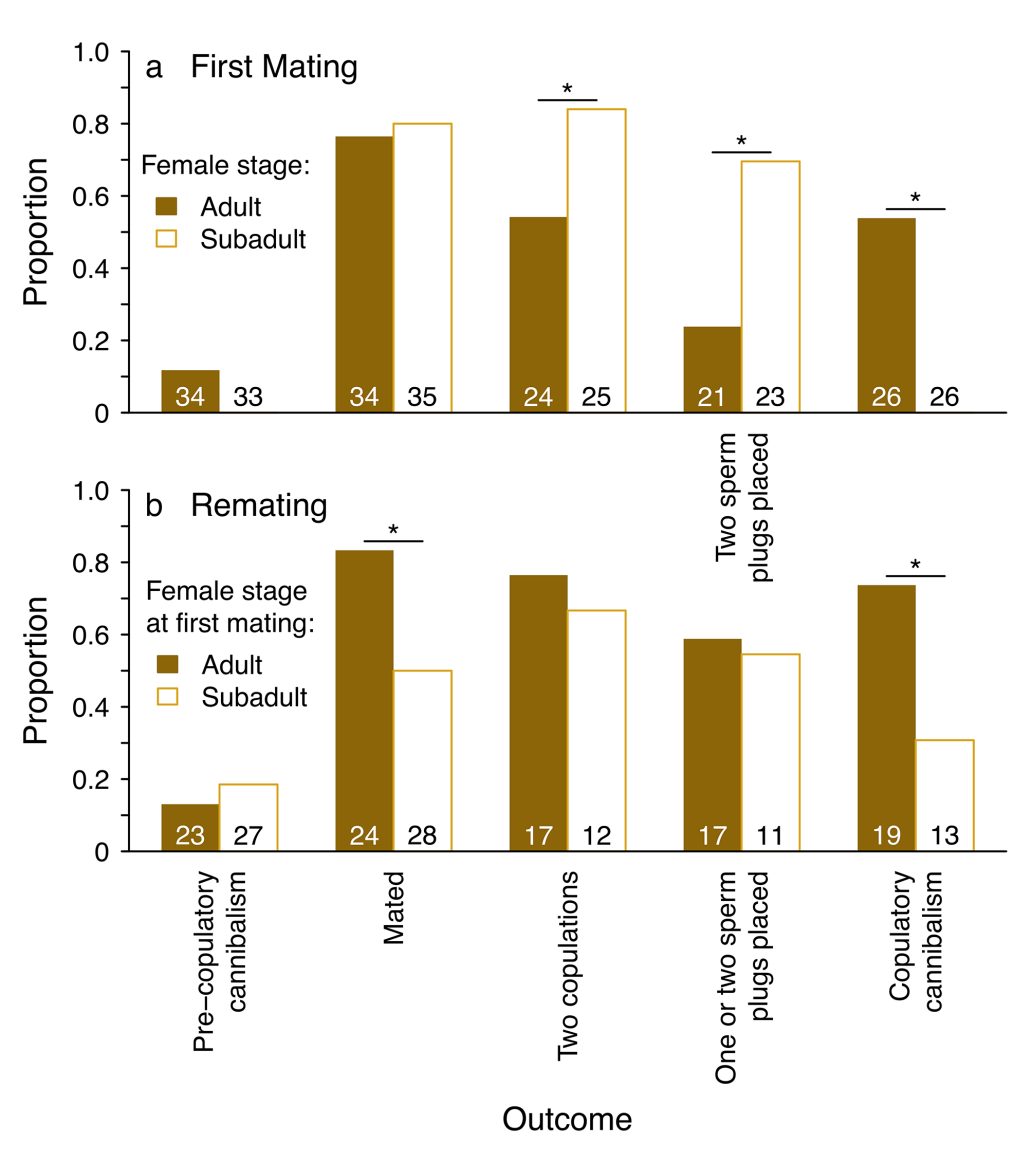
Proportions of trials in which different mating outcomes occurred when **(a)** adult or subadult females were paired with males for the first time; and **(b)** adult females previously mated as adults or previously mated as subadults were paired with a second male. Asterisks over paired bars indicate a significant difference based on a GLM or Fisher’s exact test (see text). Numbers at the x-axis within bars indicate sample sizes

When unmated females were paired with males, pre-copulatory cannibalism was rare, and occurred only in trials with adults (Fig. 1a). Males courting subadult females usually mounted the female only once prior to copulation, whereas males courting adult females dismounted and re-mounted repeatedly (χ^2^ = 79.7; *P* < 0.0001, Table 1). Adult and subadult females lunged at males equally often (χ^2^ = 0.05; *P* = 0.83; Table 1). However, adult females performed more leg flicks (χ^2^ = 9.3; *P* = 0.002) and more abdominal twitches (χ^2^ = 4.2; *P* = 0.04, Table 1).

**Table 1:** Counts of behaviours displayed by males (mounts) and females (lunges, flicks, twitches) during mating trials, reported as estimated marginal means ± SE (range), with significant differences between adult and subadult within each pairing trial type indicated in bold. The remating trials of subadult-mated females were conducted after they reached maturity *

Variable	First mating trial		Remating trial	
	Adult ♀	Subadult ♀	Adult-mated ♀	Subadult-mated ♀
n	34	35	26	28
Pre-copulatory mounts	**27.8 ± 5.9** (0–118)	**1.0 ± 0.3** (0–4)	28 ± 6 (0–64)	18 ± 4 (0–69)
Lunges	0.9 ± 0.2 (0–4)	0.9 ± 0.2 (0–5)	2.0 ± 0.5 (0–14)	1.6 ± 0.4 (0–7)
Flicks	**19.6 ± 3.2** (2–45)	**11.4 ± 2.0** (0–60)	11.8 ± 2.1 (1–55)	17.9 ± 3.2 (0–60)
Twitches	**2.6 ± 1.0** (0–20)	**0.3 ± 0.1** (0–3)	1.8 ± 0.7 (0–8)	1.2 ± 0.5 (0–7)

*statistics are reported in the text

**Fig. 2:**
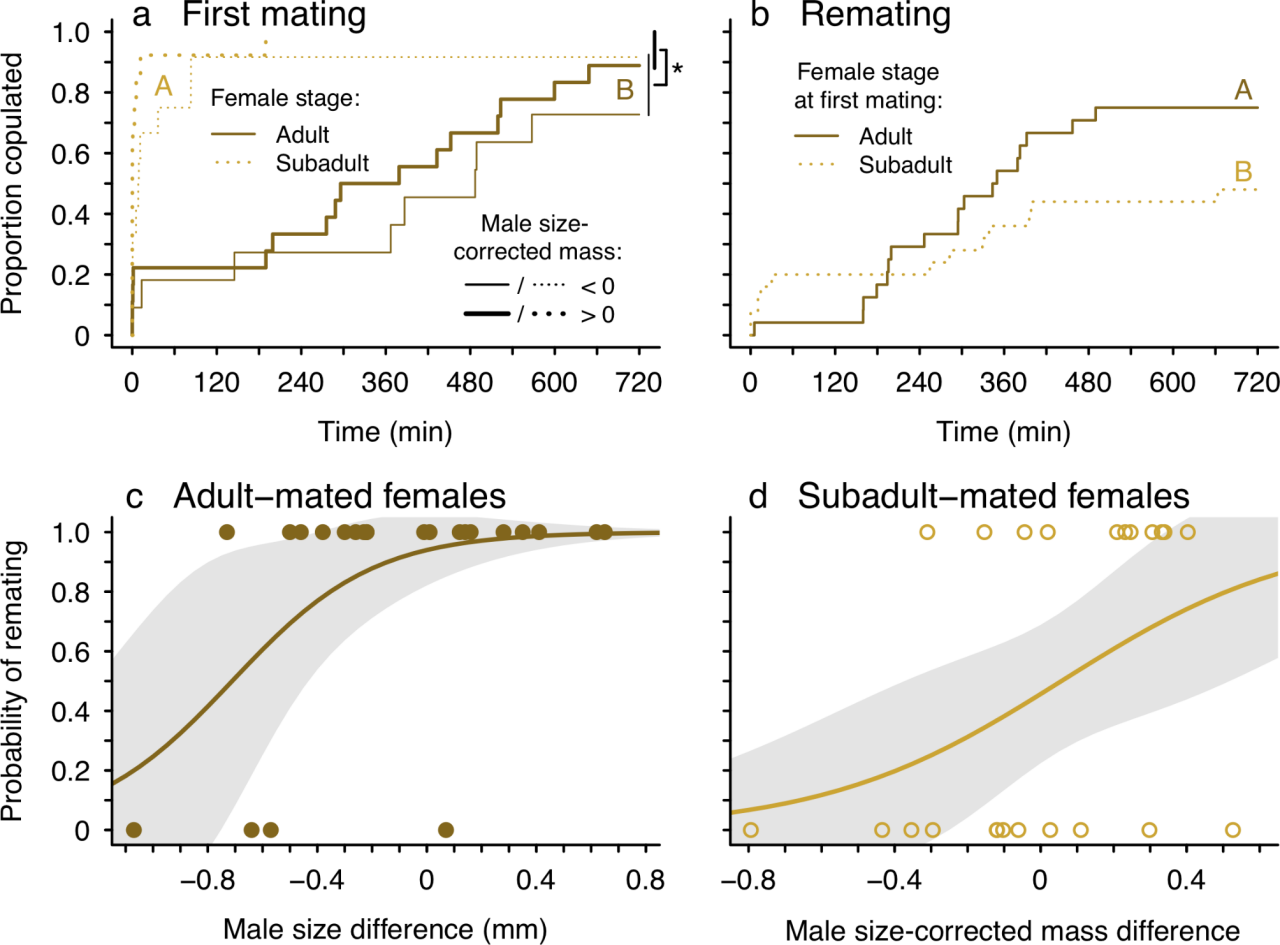
Outcomes of mating and remating trials in terms of time spent courting and the propensity of females to “trade-up” to larger males or males with greater size-corrected mass. **(a,b)** Time-to-event curves showing the latency from the first mount to the first copulation in mating trials **(a)** and remating trials **(b)** with adult (brown solid lines) and subadult (orange dotted lines) females. Curves extend to 720 min (total duration of trials) because copulation did not occur in some trials. Significant differences are indicated by different letters (female stage) or an asterisk (male size-corrected mass). Male size-corrected mass is illustrated here as a binary variable for ease of interpretation. A value > 0 indicates a mass greater than average for a given body size (heavy lines); a value of < 0 indicates mass less than average for a given body size (thin lines). **(c,d)** The relationship between the body size **(c)** or size-corrected mass **(d)** of the second-mating male relative to the first and the probability a female remates. Points **(c,d)** represent raw data, lines are predicted fits and grey areas are approximate 95% CIs from GLMs where male size-corrected mass or size difference is set at its mean for prediction

Controlling for male size and size-corrected mass, there was no difference in the latency to the first mount for males courting females from each group (χ^2^ = 0.012; *P* = 0.91). However, the latency from the first mount to the first copulation was shorter for males courting subadult females (χ^2^ = 27.2; *P* < 0.0001) and for males with greater size-corrected mass (χ^2^ = 5.25; *P* = 0.022; Fig. 2a). The probability of mating was high and did not differ for adult and subadult females (χ^2^ = 0.84; *P* = 0.36; Fig. 1a). However, most subadult females copulated twice (i.e. received two insertions) with their first mate compared to only about half of adult females (χ^2^ = 5.08; *P* = 0.024; Fig. 1a).

**Fig. 3:**
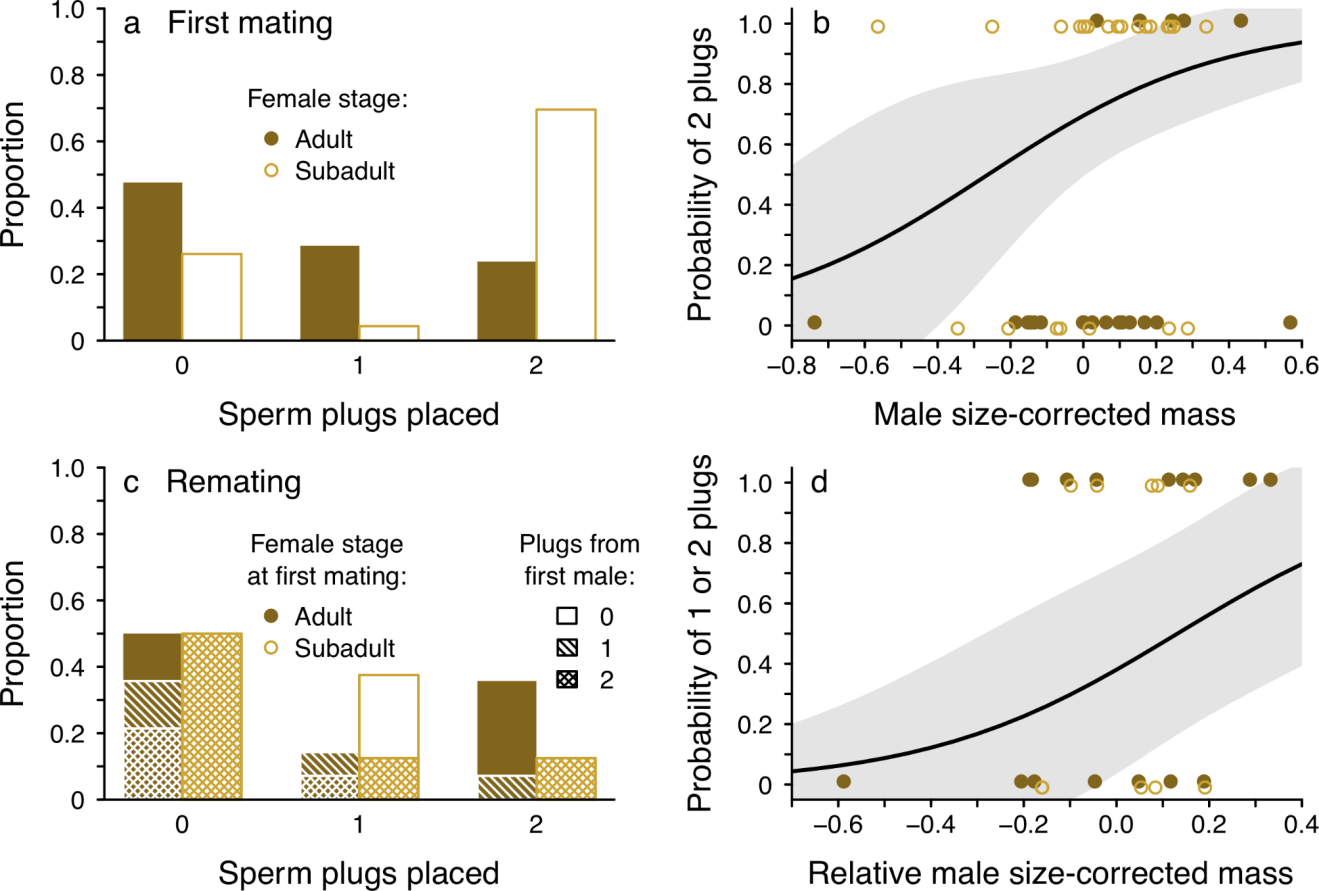
Outcome of mating and remating trials in terms of plugging success, including **(a)** the number of sperm plugs deposited in females’ paired genitalia by first-mating males; **(b)** the relationship between male size-corrected mass and plugging success (two plugs vs. one or no plugs) for first-mating males; **(c)** the number of plugs placed by second-mating males (and how many plugs were already present); and **(d)** the relationship between relative male size-corrected mass and plugging success (one or two plugs vs. no plugs) for second-mating males **(d)**. Points **(b,d)** represent raw data, lines are predicted fits and grey areas are approximate 95% CIs from GLMs where male size or size difference is set at its mean for prediction

Sperm plugs could be unambiguously assigned to males for 44 of 52 mated females. 48% of adult females (N = 21) did not have any sperm plugs in their genitalia, and only 24% of adult females had both genital tracts plugged (Figs. 1a and 3a). In contrast, most mated subadult females (N = 23) had two mating plugs in their genitalia (70%), and one plug was the least common outcome (4%; Fig. 3a). Controlling for male size, mated subadult females were more likely to have two sperm plugs than were adult females (χ^2^ = 119; *P* = 0.0008; 1a, 3a). There was also a positive relationship between plugging success and male size-corrected mass (χ^2^ = 3.8; *P* = 0.051; Fig. 3b) across trials with both subadult and adult females. Finally, subadult females never killed males whereas 54% of males that mated were killed by adult females during copulation (Fisher’s exact test: *P* < 0.0001; Fig. 1a).

### Remating trials

Pre-copulatory cannibalism was rare for previously-mated adult females in both groups (Fig. 1b), but there was an interaction between the female stage upon first mating and the size of the second male relative to the first (χ^2^ = 8.88; *P* = 0.0029). Second males that were smaller than first males were more likely to be cannibalized by adult-mated females prior to copulation (χ^2^ = 11.40; *P* = 0.0007), whereas relative male size was not related to pre-copulatory cannibalism for subadult-mated females (χ^2^ = 0.57; *P* = 0.45). There was no difference for adult-mated females compared to subadult-mated females in lunges (χ^2^ = 0.27; *P* = 0.61; Table 1), leg flicks (χ^2^ = 2.45; *P* = 0.12) nor abdominal twitches (χ^2^ = 0.49; *P* = 0.49; Table 1).

There was no difference in the latency to the first mount for males courting females from each group (χ^2^ = 0.027; *P* = 0.87). However, the latency from the first mount to the first copulation was longer for males courting subadult-mated females (χ^2^ = 4.64; *P* = 0.031), even though some males courting subadult-mated females engaged in very little proximal courtship (Fig. 2b). Males performed similar numbers of mounts when courting females from both groups (χ^2^ = 2.23; *P* = 0.14, Table 1).

Adult-mated females were more likely to remate than were subadult-mated females (main effect of female stage: χ^2^ = 7.26; *P* = 0.007; Fig. 1b), and even more so when the second male was larger than their first mate (χ^2^ = 5.86; *P* = 0.016; Fig. 2c). Although remating was unrelated to relative male body size for subadult-mated females (χ^2^ = 1.24; *P* = 0.26), these females tended to be more likely to remate when second males had greater size-corrected mass than their first mates (χ^2^ = 3.59; *P* = 0.058; Fig. 2d). Adult-mated females were more likely than subadult-mated females to engage in copulatory cannibalism of their second mate (χ^2^ = 5.33; *P* = 0.021; Fig. 1b).

There was no significant difference in the probability of copulating twice when remating for subadult-mated and adult-mated females (χ^2^ = 1.85; *P* = 0.67), nor was there any effect of relative male size (χ^2^ = 0.24; *P* = 0.62) or size-corrected mass (χ^2^ = 0.06; *P* = 0.81) on achieving two copulations (Fig. 1b).

Of males mating with both adult-mated and subadult-mated females, 50% did not place any sperm plugs. Placing two plugs was more common for males paired with adult-mated females and more likely to be effective at securing paternity; most subadult females were fully plugged by their first mates (Fig. 3c). The probability of placing at least one sperm plug was no different for adult-mated and subadult-mated females (χ2 = 0.74; P = 0.39), but males with greater size-corrected mass relative to the first mate tended to be more likely to deposit at least one plug (χ2 = 3.68.32; P = 0.055; Fig. 3d).

## Discussion

Consistent with our predictions, adult females mated indiscriminately during their first mating but employed following post-copulatory mechanisms that allowed them to ‘trade up’: these females often permitted only a single insertion with their first mates, rarely allowed males to place sperm plugs, and they readily remated, particularly when the second male was larger than the first mate. However, in contrast to our prediction that they would be choosier than adult females, subadults also mated indiscriminately at their first mating opportunity, usually allowing two insertions and placement of two sperm plugs, reducing the likelihood that subsequent mates would acquire paternity. Subadult-mated females were also much less likely to remate in their adult stage than were adult-mated females, but when they did remate, they showed signs of ‘trading up’, tending to remate with males that had greater size-corrected mass than their first mate. These results show that female use of post-copulatory choice mechanisms (i.e. insertion number, plug placement and remating) is affected by an interplay between the decisions made in the first mating and male phenotypes. Moreover, we infer that the cost of delays to reproduction may have a stronger effect on female mating decisions, and thus patterns of sexual selection, than potential benefits of choosiness related to male traits.

In *Latrodectus* spiders, females have many opportunities to either express, or ensure the opportunity to express choice [24]. This starts with behavioural responses to courting males, including aggression, mate rejection and cannibalism, all largely under female control due to extreme size dimorphism. Even if a female chooses to mate, the number of insertions she allows adjust whether the first mate will succeed or lose out in sperm competition. Her behaviour during copulation can affect successful plug placement, however, the mechanism is unknown [18]. Although the efficacy of mating plugs is unknown in *L. geometricus*, we presume that, if placed correctly, it prevents further inseminations as found in other species with similar morphology [25, 26, 28]. Finally, females can determine whether sperm competition will occur at all by choosing whether to remate. Here, we examined all of these effects simultaneously in *Latrodectus geometricus*. In our experiment, almost all adult females mated with the first male who courted them, consistent with a significant cost of rejecting a male outright [9]. There was one indication of pre-copulatory choosiness in that first mating in that the female’s latency to accept a copulation was longer for smaller males. In nature, where multiple males often arrive on the web of a signalling female [27, 32, 37], this may allow time for a superior male to usurp that first mating opportunity [38]. In the absence of an immediate alternative however, unmated adult females mated, but typically allowed only a single insertion. Only males with the greatest size-corrected mass deposited plugs in both sperm storage organs of adults allowing for first-male sperm precedence. Additionally, these adult-mated females, typically with one spermatheca empty or unplugged, then remated if the second male was larger than the first, but frequently killed smaller second males during courtship, minimizing the time wasted with males they were rejecting. Moreover, the second mates were more likely to deposit at least one sperm plug themselves when their size-corrected mass was greater than that of the first mate. Finally, since almost 80% of second matings included two copulations (compared to ~ 50% of first matings), the overall effect would be higher total insemination success and higher expected paternity of larger second males. This study echoes and extends experiments on other taxa [e.g. 39, 40] including two congeners in which females that experienced cues of low male availability as juveniles mated more indiscriminately as adults than those that experienced cues of high availability (*L. hasselti*, [18]; *L. hesperus*, [19]). While these studies only inferred that choosiness under high mate availability would favour second males, the current experiment demonstrates directly that the adult female’s mating decisions in first and second matings are linked. Assessing the seasonal or spatial variation in sex ratio or density that can shape male availability (assumed based on patterns shown in several other species, [24]) would be an important complement to this study. Overall, however, this work adds to the accumulating evidence that the ‘wallflower’ effect may be an important determinant of adult female mating behaviour across the genus [18, 24, 28].

It is less clear however, what motivates the mating and choice strategies of subadults. We predicted that subadult females encountering males would not risk much by exerting choice and potentially delaying mating, since they cannot reproduce right after mating in any case (since they must moult first). Moreover, once they become adults, their attractiveness to males increases, so they are likely to have future mating opportunities [22, 23, 35, 36]. In addition, in other *Latrodectus*, low male investment in courtship results in female discrimination (e.g., *L. hasselti*, [38]), and males approaching subadults invest minimally in courtship and do not somersault [20]. We thus expected that subadult females should be less likely to mate in ways that would cement paternity of these low-investing males. Surprisingly, subadult females mated at high rates, similar to adult females (also reported for *L. hesperus* subadults, [21]). Although fully mobile, subadults seemed generally more quiescent than adults: they did not attack and cannibalise males prior to copulation, performed fewer putative deterrent behaviours (leg flicks, abdomen twitches) towards approaching males, and rarely rebuffed male attempts to mount. Moreover, subadults accepted more copulations during their first mating than adults, and the majority of them bore plugs in both spermathecae. The only indication of choosiness in subadults was that the few males that failed to deposit two plugs had lower size-corrected mass than other males. Overall, in the majority of cases, the patterns of mating outcomes would effectively prevent subadult females from ‘trading up’ through mating with another male.

We consider two possible ways in which males that attempt to mate with subadults may reliably be high quality males in nature, and thus subadults that mate indiscriminately may benefit from these mating patterns. Since males typically engage only in very reduced courtship with subadult females [20, 35, 36], the male’s presence in the female’s web may be the only information available about his quality. First, despite the lower attractiveness of subadult silk extract (*L. hesperus*, [23]), and of subadults in their webs in laboratory trials [22, 35], in several widow spider species males nevertheless locate subadult females and live on their webs for days prior to attempting a mating in nature [27, 32]. During this ‘cohabitation’ period, males of some *Latrodectus* species aggressively deter rivals from the web (‘mate guarding’; e.g., *L. hesperus*, C. Scott, pers. obs.). If this is the case for *L. geometricus*, then subadults will typically mate with competitively superior males [e.g., 41]. Second, males that manage to locate a subadult despite a lack of strong pheromonal cues may be those that are more efficient at mate searching, a challenge that results in high mortality rates in nature for many *Latrodectus* species [27, 33]. Thus, inter-male competition, either through contests or mate searching may be the primary selective force determining which males successfully mate with subadult females, whereas female choice plays a more important role in determining male mating success with adult females.

Regardless of why subadult females are not choosy in their first mating opportunity, this unexpected first-mating pattern has implications for female behaviour in second matings. Since more than 60% of subadults had two sperm plugs in place, the opportunity to shift paternity through a second mating was relatively low. Consistent with this, only ~ 40% of subadult-mated females remated with a second male (about half the remating rate of adults) and these females may have been more reluctant to remate than adults (latency to remating was longer). It is likely that, in nature, remating rates of subadult-mated females would be even lower than those measured here, since these females are less likely to attract males than adults. For example, in a congener, silk extracts from adult females that mated as subadults triggered highly variable, but overall lower male activity than was the case for females mated as adults (*L. hasselti*, [23]). For *L. geometricus* females in this experiment, not only were subadult-mated females less likely to remate, they were also more choosy than they had been in their first mating. The relatively few subadults that did remate were more likely to do so with males of greater size-corrected mass than their first mate. Additionally, the second mates were more likely to deposit at least one sperm plug (although this would not effectively enhance paternity if the female was already plugged) when their size-corrected mass was greater than that of the first mate.

The differences in choosiness we have demonstrated have implications for selection on male mating investment, mate searching, and mate choice. Previous studies showing low costs and high mating success associated with immature mating [20, 35] led to predictions that males should prefer to approach and mate with subadults over adult females, however these have not been supported [22, 35, 36]. Our results suggest additional benefits of immature mating: in addition to sperm-precedence for the first-mating male and high sperm plugging success, subadult females rarely remate, further limiting the risk of sperm competition. Therefore, for males, investing in subadult females through other paternity assurances such as prolonged courtship or somersaulting, may be unnecessary. It remains puzzling that males nevertheless express strong preferences for adult females. This may arise from the advantage of mating with a female who is ready to produce offspring, rather than risking the possibility of reproductive failure if a subadult female dies during the final moult, or has low foraging success as an adult. We rarely observed females to die during moulting under laboratory conditions, however, such mortality can be significant under natural conditions since the moulting process is a sensitive period due to the risk of predation on moulting or freshly moulted spiders, the risk of desiccation or an inability to release the old cuticle (e.g. [42–44]). Alternatively, males may rarely have a choice between subadult and adult females in the field, because widows are protandrous and cohabitation with subadults is common [24]. If most females are mated as subadults, males will only rarely have the option to approach and mate with signaling adult females. Whereas about one third of females appear to have been mated as subadults in one field population of *L. hasselti* [20], almost all females appear to mate as subadults, rather than as adults, in one population of *L. hesperus* (C. Scott, pers. obs.). More detailed investigations of mating systems in the field are necessary to determine whether this tactic is rare or common in nature, and what ecological factors are linked to its frequency. Such data will untangle the extent to which immature mating is a common female mating outcome, and whether it is a purely opportunistic male tactic.

## Conclusions

Here we have shown support for theoretical predictions related to pre- and postcopulatory mating decisions by females who mate as adults, but unexpected mating decisions by females who mate as subadults. This study highlights how female mate choice and choosiness can shift across developmental contexts. Moreover, these results support an expanding appreciation of the extent to which female mating decisions across their lifetimes can be interconnected, and strongly related to risks of delays to mating, rather than primarily related to male phenotypes [9].

## Methods

### Experimental spiders

Adult females of *Latrodectus geometricus* C. L. Koch, 1841 (N = 24) were collected in Los Angeles, California, USA (34.11023591671581, -118.29157616275369) in January and December 2020 from spatially separated webs. Females were transported to the laboratory at the University of Toronto Scarborough and reared in a temperature-controlled room at 25 °C (similar to the mean temperature during the summer months in Los Angeles) and on a 12:12 h light:dark cycle. Females were held in clear plastic containers (5 × 5 × 7 cm). Egg sacs were removed as they were produced and placed into a larger clear plastic container (9 × 9 × 11.5 cm). After emerging, spiderlings were kept together until their second moult, after which they were transferred into separate containers. Spiderlings and adult males were fed fruit flies twice per week. Females were fed weekly with one cricket (*Acheta domesticus or Gryllodes sigillatus*) once they reached a size typical of the fifth instar. We monitored males and females and recorded the dates of their moults.

We used spiders from the first laboratory-reared generation for our experiments. Adult females and adult males are readily recognized by their mature genital morphology. Subadult females were only used no earlier than 6 days before the final moult because only then they are capable of mating and storing sperm [20, 31, 36]. These late-subadults are identified by the visible swelling of the region of exoskeleton covering the developing external genitalia, and a colour change from grey to dark brown [20]. After trials, the date of subadult’s final moult was recorded to allow post- hoc assessment of age. In our mating and remating trials, females were never paired with males from the same family line, and the two males paired with a given female originated from a different family line.

### Experimental setup

Adult and late-subadult females were placed in mating arenas for 48 h to construct webs prior to the mating trials. Mating arenas consisted of a plastic block (11 × 8 × 8 cm) holding two inverted U-shaped metal frames attached in parallel (used by females as web attachment points), and placed inside an experimental container (35 × 30 × 15 cm) with water to prevent escape of the spiders (also used in [21, 23, 35]).

Due to lengthy courtship observed in a previous study [35] the spiders were allowed to court and mate for 12 h during mating and remating trials. Spiders were recorded for these 12 h on digital video using Panasonic low-light black and white cameras (WV BP330) with macro zoom lenses (Navitar Macro-Zoom 7000) under low-lux red-light illumination. All experiments were conducted during the dark phase under a red light since these spiders are nocturnal [45]. Females were weighed using an analytical balance (Ohaus electronic balance, accurate to 0.01 mg) at the end of the second trial. Males were weighed before the start of trials due to the risk of sexual cannibalism during the mating.

After the mating trials, males and females were euthanized by freezing and fixed in 70% ethanol. Males were then photographed under a dissecting microscope and the mean tibia-patella length of their forelegs was measured using imageJ. The residuals from a regression of log(male mass) against tibia-patella length were used to calculate size-corrected mass (an index of body condition [46]).

### Sub-adult mating and remating

We paired unmated late-subadult females that were capable of mating [31, 35, 36] (N = 39; median age: 3 days prior to adulthood; range 0–6 days) with unmated adult males (N = 39; median age: 16 days post maturity; range: 6–21 days). If the subadult females mated (N = 28), they were kept on the same web and, after they moulted to adulthood, they were paired with another unmated male (N = 28; median age: 17 days; range: 12–21 days) for a remating trial which occurred 5–13 days after the first mating (median time after the adult moult: 5 days, range 4–9 days). Females were not used if they produced an eggsac before the second pairing.

### Adult mating and remating

Unmated adult females (N = 34; median age: 5 days after their final moult; range 4–8 days) were paired with unmated adult males (N = 34; median age: 18 days; range: 13–21 days). Adult females were chosen to ensure their median age at their first pairing was comparable to that of subadult-mated females during their adult remating trials. If adult females mated (N = 25), they were kept on the same web and 4–10 days later they were paired with a new unmated adult male (N = 25; median age: 16 days; range: 4–30 days) for remating trials. The median age of adult-mated females during the remating trials was 13 days (range: 9–18 days). Females were not used if they produced an eggsac before the second pairing.

### Sperm plugs

Pedipalps of mated males were checked under a dissecting microscope to establish whether males lost the tips of their copulatory organs (apical sclerites). The reproductive tract (spermathecae and copulatory ducts) of mated females were dissected and macerated at room temperature in 10% KOH for 4 days, then preserved in 70% ethanol. The transparent genital tract was then inspected for sclerites under a dissecting microscope. By comparing male sclerite loss and female genitalia, we were able to unambiguously assign sclerites to males in all but eight cases. For example, if the first male lost both sclerites and the second male lost one, and the female’s genitalia contained three sclerites in total, two could be assigned to the first male and one to the second. However, if each male lost both sclerites and the female’s genitalia contained three sclerites in total, it is not clear whether the first male placed two and the second male one, or vice versa. Multiple sclerites can be lodged in the same spermatheca; only sclerites placed in the opening to the spermatheca function as sperm plugs and block insemination [24].

### Video analyses

One of us (LS) scored the videos and recorded the progression and outcome of mating and males’ and females’ behaviours that reflect established stages of mating interactions [21, 23]. We recorded whether females attacked and killed males before copulation (‘pre-copulatory cannibalism’). During mating interactions, in several *Latrodectus* species, female movements and direct contact with the male with their forelegs (e.g., ‘strikes’) have been recorded and may function to deter male courtship and mating attempts [23]. For the first hour after introduction of the male when deterrent behaviours are most common (Sentenská, pers. obs.), we counted the number of following putative deterrent behaviours by females: (1) ‘lunges’ when the female rapidly approached the male in a manner similar to predation behaviour; (2) ‘abdominal twitches’ represented by vigorous vibrations of the female’s abdomen in response to an approaching male and (3) ‘foreleg strikes’: rapid flicking movements of the female’s front legs when touched by the male [23, 47]. Further, throughout the whole recordings lasting 12 hours, we quantified components of male courtship in terms of how much time the male invested in (1) ‘distal courtship’ [21, 23]: latency from the start of the trial to the first mount (defined as when the male first moves onto the female’s ventral abdominal surface, which is the location of the female’s genital openings), and (2) ‘proximal courtship’ [21, 23, 48–50]: latency from the first mount until the copulation. We also counted the number of mounts males performed before the first copulation [23] or, if copulation did not occur, the number of mounts during the recorded 12 hours as another indicator of courtship effort. In all mating and remating trials a mating was scored as successful if the male copulated at least once, and we counted the number of copulations achieved (at least 2 are required to inseminate both spermathecae).

### Statistical analyses

We ran all analyses in R (version 4.2.1; [51], for code see [52]). We compared courtship behaviour and mating outcomes for first mating trials and remating trials. Despite randomly assigning males to treatment groups, males paired with unmated subadult females had significantly longer legs than males in all other mating trials (*F* = 7.87; df = 3; *P* < 0.0001; Figure S1). There was no difference in male size-corrected mass across treatments (*F* = 0.18; df = 3; *P* = 0.91). We controlled for differences in male size and size-corrected mass by including both of these measures as covariates of all analyses. Our analyses used cox-proportional hazards models (survival analysis) for latencies (R package “survival” [53, 54]), generalized linear models (GLMs) with negative binomial distribution and log link for counts (R package “MASS”, [55]), and GLMs with the binomial distribution and logit link for binary outcomes (R package “stats”, [51]). In all models, we first included fixed effects of female stage and its interactions with male size and size-corrected mass (first mating trials) or with the difference betweenthe size and size-corrected mass of the first and second mate (remating trials). We then used the Anova() function in R (R package “car”; [51]) to produce type-II analysis-of-variance tables to assess the significance of each term in the model. If the interaction terms were not significant at α = 0.05, we re-ran the model with only the main effects. When there were significant interactions, we made additional models assessing the effects of the same predictors for each female stage separately. We report likelihood-ratio χ^2^ statistics and p-values from Anova tables for predictor effects in the results. Detailed statistical methods and results, including effect sizes for predictors in each final model are reported in the Additional material [see Additional file]. To assess the potential for trading up after first matings, we compared cases where the female’s first mate placed two plugs (which would block insemination by rivals), to cases where the first mate achieved one or no plugs, which would allow second mates to acquire paternity. Moreover, we assessed whether second males that successfully mated were able to deposit at least one plug, which would likely enhance their paternity in cases where the first male placed only one plug (or zero).

## Electronic supplementary material

Below is the link to the electronic supplementary material.

Supplementary Material 1

## Data Availability

The dataset generated during the current study is available in the Dryad repository [https://datadryad.org/stash/share/VKTYxjKrFd3u4bmS9RDQ2jv84Zg5_QkDCG6kr7libjs]^52^.
