# Supplementary material for: Pre-copulatory choices drive post-copulatory decisions: mechanisms of female control shift across different life stages
Source: BMC Ecol Evol. 2023 Jun 27;23:25. doi: 10.1186/s12862-023-02138-6 (PMC10294400; doi:10.1186/s12862-023-02138-6)
Supplement: Supplementary file 1 — Supplementary Material 1 [file 12862_2023_2138_MOESM1_ESM.docx]

Additional file for

**Pre-copulatory choices drive post-copulatory decisions: Mechanisms of female control shift across different life stages**

Lenka Sentenská^1,2^, Catherine Scott^2,3^, Luciana Baruffaldi^2^, Maydianne C. B. Andrade^2^

^1^ Department of General and Systematic Zoology, University of Greifswald, Loitzer Strasse 26, Greifswald, Germany 17489

^2^ Department of Biological Sciences, University of Toronto Scarborough, 1265 Military Trail, Toronto, Ontario, Canada M1C 1A4

^3^ Department of Natural Resource Sciences, McGill University, 21111 Lakeshore Road

Ste-Anne-de-Bellevue, Québec, Canada H9X 3V9

Supplementary Methods

***Male size and assignment to treatment group***

Despite randomly assigning males to treatment groups, there were differences in male body size (leg lengths) across treatments (Anova: *F* = 7.87; df = 3; *P* <0.0001). Males assigned to mate with subadult females (first pairings) were significantly larger than males assigned to mate with adult females and subadult-mated females (Tukey’s HSD; Figure S1).


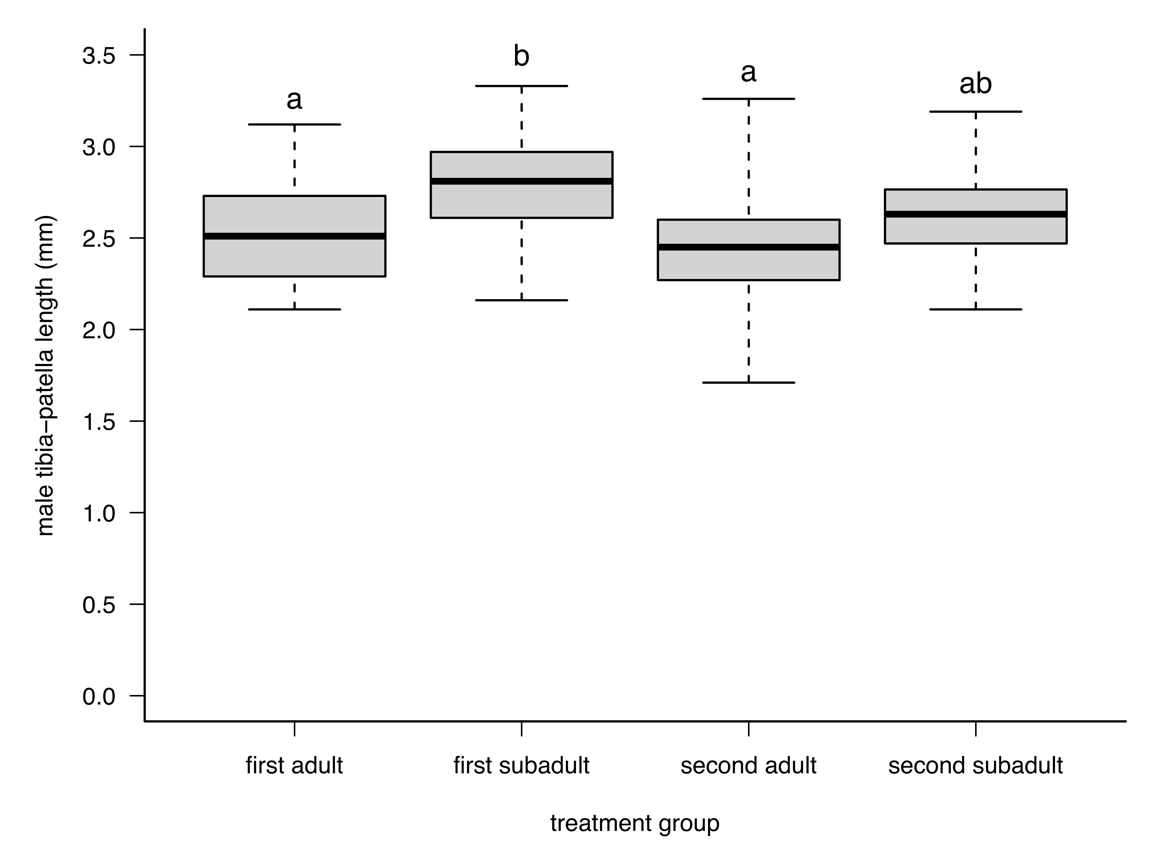


**Figure S1.** Boxplots of the body size (leg length) of males randomly assigned to mate with females in each treatment group. Different letters indicate significant differences.

***Statistical methods***

We ran GLMs with appropriate distributions for each response variable: negative binomial for count data; binomial (logistic regression) for binary outcomes. We ran Cox-proportional hazards models for latencies to events. For analyses of pre-copulatory behaviours in the first round of mating trials, we began by including the following variables as fixed effects in models: the interaction of female stage (subadult vs. adult) with male size-corrected mass (calculated as residuals from a the regression of log(male mass) against tibia-patella length) and male body size (tibia-patella length). For analyses of remating trials, we began by including the following variables as fixed effects in models: female stage at first mating (subadult vs. adult) and its one-way interactions with both relative male size-corrected mass and relative body size (calculated as the size-corrected mass or leg length, respectively, of the second-mating male subtracted from that of the first-mating male). We then used likelihood ratio tests to assess whether interaction terms were significant at α = 0.05. If there were no significant interactions, we re-ran models without these terms. If there were significant effects of the interaction between female stage and one or more other predictors, we ran additional models to assess the effects of individual predictors for subadult (or subadult-mated) and adult (or adult-mated) females separately. We report likelihood ratio χ^2^ statistics and p-values for each predictor in the main text. Below we report detailed summaries of all final models, including effect sizes. All data and R code are available as supplementary material and will be deposited to Dryad before publication.

Supplementary Results

***First pairing: unmated adult vs. subadult females***

1. Number of pre-copulatory mounts.

**Table S1.** Output from a negative binomial GLM with number of mounts prior to copulation as the response variable.

| *Predictors* | *Incidence Rate Ratios* | *CI* | *p* |
| --- | --- | --- | --- |
| (Intercept) | 7.81 | 0.33 – 201.39 | 0.176 |
| **female stage [subadult]** | **0.04** | **0.02 – 0.07** | **<0.001** |
| male leg length | 1.60 | 0.47 – 5.48 | 0.423 |
| size-corrected mass | 2.91 | 0.54 – 15.96 | 0.139 |
| Observations | 65 | | |
| R^2^ Nagelkerke | 0.818 | | |

2. Number lunges by females toward courting males.

**Table S2.** Output from a negative binomial GLM with number of lunges prior to the first mount as the response variable.

| *Predictors* | *Incidence Rate Ratios* | *CI* | *p* |
| --- | --- | --- | --- |
| (Intercept) | 0.12 | 0.01 – 2.02 | 0.141 |
| female stage [subadult] | 0.93 | 0.47 – 1.81 | 0.823 |
| male leg length | 2.19 | 0.73 – 6.79 | 0.159 |
| size-corrected mass | 0.37 | 0.10 – 1.28 | 0.135 |
| Observations | 65 | | |
| R^2^ Nagelkerke | 0.114 | | |

3. Number abdominal twitches by females.

**Table S3.** Output from a negative binomial GLM with number of twitches prior to the first mount as the response variable.

| *Predictors* | *Incidence Rate Ratios* | *CI* | *p* |
| --- | --- | --- | --- |
| (Intercept) | 0.15 | 0.00 – 144.98 | 0.496 |
| **female stage [subadult]** | **0.10** | **0.02 – 0.44** | **0.001** |
| male leg length | 2.89 | 0.20 – 55.30 | 0.321 |
| size-corrected mass | 0.12 | 0.00 – 2.29 | 0.101 |
| Observations | 65 | | |
| R^2^ Nagelkerke | 0.310 | | |

4. Number of leg flicks by females.

**Table S4.** Output from a negative binomial GLM with number of flicks prior to the first mount as the response variable.

| *Predictors* | *Incidence Rate Ratios* | *CI* | *p* |
| --- | --- | --- | --- |
| (Intercept) | 11.95 | 1.40 – 105.12 | 0.021 |
| **stage [subadult]** | **0.58** | **0.35 – 0.98** | **0.030** |
| male leg length | 1.20 | 0.52 – 2.81 | 0.655 |
| size-corrected mass | 0.84 | 0.34 – 2.05 | 0.732 |
| Observations | 65 | | |
| R^2^ Nagelkerke | 0.096 | | |

5. Latency to first mount (duration of distal courtship on the web).

**Table S5.** Output from a Cox proportional hazards model with latency to first mount as the response variable.

| *Predictors* | *Hazard Ratios* | *CI* | *p* |
| --- | --- | --- | --- |
| female stage [subadult] | 1.04 | 0.55 – 1.95 | 0.912 |
| male leg length | 0.50 | 0.18 – 1.38 | 0.181 |
| male condition index | 0.80 | 0.26 – 2.50 | 0.707 |
| Observations | 64 | | |
| R^2^ Nagelkerke | 0.035 | | |

6. Latency from the first mount to the first copulation (duration of proximal courtship on and near the female’s body).

**Table S6.** Output from a Cox proportional hazards model with latency from the first mount to the first copulation as the response variable.

| *Predictors* | *Hazard Ratios* | *CI* | *p* |
| --- | --- | --- | --- |
| **female stage [subadult]** | **7.44** | **3.41 – 16.20** | **<0.001** |
| male leg length | 0.38 | 0.13 – 1.11 | 0.076 |
| **size-corrected mass** | **5.28** | **1.27 – 21.98** | **0.022** |
| Observations | 54 | | |
| R^2^ Nagelkerke | 0.408 | | |

7. Pre-copulatory cannibalism by adult females.

**Table S7.** Output from a logistic regression with the occurrence of pre-copulatory cannibalism as the response variable for adult females only (subadult females never engaged in pre-copulatory cannibalism).

| *Predictors* | *Odds Ratios* | *CI* | *p* |
| --- | --- | --- | --- |
| (Intercept) | 0.11 | 0.00 – 3770.28 | 0.673 |
| male leg length | 1.05 | 0.02 – 59.34 | 0.982 |
| size-corrected mass | 0.09 | 0.00 – 7.51 | 0.291 |
| Observations | 33 | | |
| R^2^ Tjur | 0.03 | | |

8. Mating success.

**Table S8.** Output from a logistic regression with the occurrence of mating (at least one copulation) as the response variable.

| *Predictors* | *Odds Ratios* | *CI* | *p* |
| --- | --- | --- | --- |
| (Intercept) | 219.28 | 0.80 – 118345.27 | 0.072 |
| female stage [subadult] | 1.82 | 0.51 – 6.95 | 0.364 |
| male leg length | 0.19 | 0.02 – 1.67 | 0.148 |
| size-corrected mass | 2.86 | 0.21 – 45.43 | 0.433 |
| Observations | 67 | | |
| R^2^ Tjur | 0.046 | | |

9. Likelihood of two copulations vs. only one.

**Table S9.** Output from a logistic regression with the number of copulations (two vs. one) as the response variable.

| *Predictors* | *Odds Ratios* | *CI* | *p* |
| --- | --- | --- | --- |
| (Intercept) | 0.02 | 0.00 – 15.76 | 0.259 |
| **female stage [subadult]** | **5.49** | **1.24 – 30.99** | **0.033** |
| male leg length | 4.55 | 0.35 – 69.51 | 0.254 |
| size-corrected mass | 2.93 | 0.09 – 107.18 | 0.544 |
| Observations | 47 | | |
| R^2^ Tjur | 0.181 | | |

10. Sperm plugging success. Two correctly-placed plugs are required to block insemination by rivals.

**Table S10.** Output from a logistic regression with sperm plugging success (two plugs placed vs. one or none) as the response variable.

| *Predictors* | *Odds Ratios* | *CI* | *p* |
| --- | --- | --- | --- |
| (Intercept) | 4.31 | 0.00 – 11181.92 | 0.698 |
| **female stage [subadult]** | **12.27** | **2.92 – 69.64** | **0.003** |
| male leg length | 0.10 | 0.01 – 1.08 | 0.438 |
| **size-corrected mass** | **34.54** | **1.79 – 1117.41** | **0.069** |
| Observations | 44 | | |
| R^2^ Tjur | 0.287 | | |

***Second pairing (remating): adult-mated vs. subadult-mated females***

11. Number of pre-copulatory mounts.

**Table S11.** Output from a negative binomial GLM with number of mounts prior to copulation as the response variable.

| *Predictors* | *Incidence Rate Ratios* | *CI* | *p* |
| --- | --- | --- | --- |
| (Intercept) | 29.20 | 19.74 – 45.57 | <0.001 |
| female stage [subadult-mated] | 0.64 | 0.35 – 1.15 | 0.129 |
| male size difference | 1.35 | 0.57 – 3.16 | 0.443 |
| size-corrected mass difference | 1.58 | 0.63 – 3.72 | 0.341 |
| Observations | 47 | | |
| R^2^ Nagelkerke | 0.103 | | |

12. Number lunges by females toward courting males.

**Table S12.** Output from a negative binomial GLM with number of lunges prior to the first mount as the response variable.

| *Predictors* | *Incidence Rate Ratios* | *CI* | *p* |
| --- | --- | --- | --- |
| (Intercept) | 2.08 | 1.23 – 3.65 | 0.005 |
| female stage [subadult-mated] | 0.82 | 0.38 – 1.75 | 0.596 |
| male size difference | 1.96 | 0.68 – 5.85 | 0.173 |
| size-corrected mass difference | 0.52 | 0.16 – 1.64 | 0.298 |
| Observations | 47 | | |
| R^2^ Nagelkerke | 0.098 | | |

13. Number abdominal twitches by females.

**Table S13.** Output from a negative binomial GLM with number of twitches prior to the first mount as the response variable.

| *Predictors* | *Incidence Rate Ratios* | *CI* | *p* |
| --- | --- | --- | --- |
| (Intercept) | 1.76 | 0.83 – 4.34 | 0.139 |
| female stage [subadult-mated] | 0.65 | 0.19 – 2.19 | 0.430 |
| male size difference | 0.73 | 0.17 – 2.77 | 0.658 |
| size-corrected mass difference | 3.01 | 0.26 – 39.38 | 0.216 |
| Observations | 47 | | |
| R^2^ Nagelkerke | 0.037 | | |

14. Number of leg flicks by females.

**Table S14.** Output from a negative binomial GLM with number of flicks prior to the first mount as the response variable.

| *Predictors* | *Incidence Rate Ratios* | *CI* | *p* |
| --- | --- | --- | --- |
| (Intercept) | 12.33 | 8.76 – 17.93 | **<0.001** |
| female stage [subadult-mated] | 1.52 | 0.90 – 2.58 | 0.099 |
| male size difference | 1.63 | 0.79 – 3.38 | 0.144 |
| size-corrected mass difference | 0.91 | 0.39 – 2.01 | 0.818 |
| Observations | 47 | | |
| R^2^ Nagelkerke | 0.101 | | |

15. Latency to first mount (duration of distal courtship on the web)

**Table S15.** Output from a Cox proportional hazards model with latency to first mount as the response variable.

| *Predictors* | *Hazard Ratios* | *CI* | *p* |
| --- | --- | --- | --- |
| female stage [subadult-mated] | 1.05 | 0.57 – 1.93 | 0.869 |
| male size difference | 1.65 | 0.75 – 3.65 | 0.213 |
| size-corrected mass difference | 0.60 | 0.21 – 1.73 | 0.346 |
| Observations | 48 | | |
| R^2^ Nagelkerke | 0.057 | | |

16. Latency from the first mount to the first copulation (duration of proximal courtship on and near the female’s body).

**Table S16.** Output from a Cox proportional hazards model with latency from the first mount to the first copulation as the response variable.

| *Predictors* | *Hazard Ratios* | *CI* | *p* |
| --- | --- | --- | --- |
| **female stage [subadult-mated]** | **0.42** | **0.18 – 0.94** | **0.036** |
| male size difference | 0.97 | 0.38 – 2.49 | 0.952 |
| size-corrected mass difference | 2.34 | 0.53 – 10.38 | 0.264 |
| Observations | 45 | | |
| R^2^ Nagelkerke | 0.111 | | |

17. Mating success.

**Table S17.** Output from a logistic regression with the occurrence of mating (at least one copulation) as the response variable.

| *Predictors* | *Odds Ratios* | *CI* | *p* |
| --- | --- | --- | --- |
| (Intercept) | 16.21 | 3.16 – 286.52 | 0.010 |
| **female stage [subadult-mated]** | **0.05** | **0.00 – 0.32** | **0.009** |
| male size difference | 42.52 | 1.59 – 8954.38 | 0.068 |
| size-corrected mass difference | 2.83 | 0.31 – 36.36 | 0.379 |
| **stage [subadult-mated] ***  **male size difference** | **0.00** | **0.00 – 0.33** | **0.029** |
| Observations | 46 | | |
| R^2^ Tjur | 0.284 | | |

18. Mating success for adult-mated females.

**Table S18.** Output from a logistic regression with the occurrence of mating by adult-mated females only (at least one copulation) as the response variable.

| *Predictors* | *Odds Ratios* | *CI* | *p* |
| --- | --- | --- | --- |
| (Intercept) | 14.49 | 2.90 – 256.68 | 0.012 |
| **male size difference** | **47.93** | **1.89 – 9316.07** | **0.056** |
| size-corrected mass difference | 0.22 | 0.00 – 7.94 | 0.431 |
| Observations | 23 | | |
| R^2^ Tjur | 0.354 | | |

19. Mating success for subadult-mated females.

**Table S19.** Output from a logistic regression with the occurrence of mating by subadult-mated females only (at least one copulation) as the response variable.

| *Predictors* | *Odds Ratios* | *CI* | *p* |
| --- | --- | --- | --- |
| (Intercept) | 0.72 | 0.24 – 1.88 | 0.511 |
| male size difference | 0.20 | 0.01 – 3.35 | 0.281 |
| **size-corrected mass** **difference** | **21.55** | **0.91 – 1325.24** | **0.091** |
| Observations | 23 | | |
| R^2^ Tjur | 0.227 | | |

20. Likelihood of two copulations vs. only one.

**Table S20.** Output from a logistic regression with the number of copulations (two vs. one) as the response variable.

| *Predictors* | *Odds Ratios* | *CI* | *p* |
| --- | --- | --- | --- |
| (Intercept) | 3.08 | 1.04 – 11.61 | 0.060 |
|  |  |  |  |
| female stage [subadult-mated] | 0.64 | 0.07 – 5.16 | 0.667 |
| male size difference | 0.51 | 0.03 – 7.84 | 0.625 |
| size-corrected mass difference | 1.60 | 0.03 – 78.10 | 0.809 |
| Observations | 26 | | |
| R^2^ Tjur | 0.010 | | |

21. Likelihood of copulatory cannibalism.

**Table S21.** Output from a logistic regression with the occurrence of copulatory cannibalism (yes or no) as the response variable.

| *Predictors* | *Odds Ratios* | *CI* | *p* |
| --- | --- | --- | --- |
| (Intercept) | 2.76 | 1.01 – 9.13 | 0.064 |
|  |  |  |  |
| **female stage [subadult-mated]** | **0.11** | **0.01 – 0.73** | **0.034** |
| male size difference | 0.64 | 0.04 – 8.33 | 0.732 |
| size-corrected mass difference | 2.52 | 0.06 – 123.21 | 0.620 |
| Observations | 29 | | |
| R^2^ Tjur | 0.20 | | |

22. Sperm plugging success for second-mating males.

**Table S22.** Output from a logistic regression with sperm plugging success (one or two plugs placed vs. zero) as the response variable.

| *Predictors* | *Odds Ratios* | *CI* | *p* |
| --- | --- | --- | --- |
| (Intercept) | 1.35 | 0.47 – 4.10 | 0.584 |
| female stage [subadult] | 0.46 | 0.06 – 2.67 | 0.399 |
| **size-corrected mass** | **40.96** | **0.93 – 5022.36** | **0.082** |
| Observations | 26 | | |
| R^2^ Tjur | 0.133 | | |

23. Copulatory cannibalism.

**Table S23.** Output from a logistic regression with the occurrence of copulatory cannibalism as the response variable.

| *Predictors* | *Odds Ratios* | *CI* | *p* |
| --- | --- | --- | --- |
| (Intercept) | 2.76 | 1.01 – 9.13 | 0.064 |
| **female stage [subadult-mated]** | **0.11** | **0.01 – 0.73** | **0.034** |
| male size difference | 0.64 | 0.04 – 8.33 | 0.732 |
| size-corrected mass difference | 2.52 | 0.06 – 123.21 | 0.620 |
| Observations | 29 | | |
| R^2^ Tjur | 0.200 | | |
